# The Red Cross Society

**Published:** 1898-10-29

**Authors:** 


### THE RED CROSS SOCIETY.

The Red Cross Society's steamer "Mayflower" which was chartered by the National Society for Aid to Sick and Wounded in War (the British Red Cross Society) to convey sick and wounded soldiers from the Soudan between Assouan and Cairo is a stern-paddle steamer 140 feet long. She carried two medical officers and eight men of the Royal Army Medical Corps, lent by the Government, and two nurses sent out by the society. Reconstructed from a pleasure vessel into a hospital ship to hold 52 sick and wounded, and, on an emergency, 72 patients, she was fitted out with the most modern medical and surgical appliances and medical comports of every description. The sick and wounded thus enjoyed the luxury of being conveyed by river, and were saved the heat, choking dust, and severe discomforts of the railway journey of about 600 miles between Assouan and Cairo, which a high authority has described as the worst part of the transit from Khartoum.

We are informed that Lord Wantage, the chairman of the Council of the National Society for Aid to the Sick and Wounded in War (British Red Cross Society), has received from Colonel Young, the society's commissioner, a telegraphic communication from Cairo, in which he states that, after conferring with Lord Cromer and the Sirdar as to the best means of showing in a practical form the society's sympathy with all sufferers from wounds or sickness in the recent operations in the Soudan irrespective of nationality, he has placed in the Sirdar's hands the sum of £300 to be applied directly for the benefit of the Egyptian soldiers who have suffered in the campaign, and the sum of £200 for the purchase and immediate dispatch to Omdurman of articles urgently needed for the Dervish wounded, who are being treated by the Egyptian medical staff. Lord Wantage and the Council of the Red Cross Society have given their sanction to this application of the funds. The society's steamer, "Mayflower," returned to Assouan on the 3rd inst. to bring down the last of the sick from the front. This voyage terminates the society's work of evacuating the sick and wounded, and the society's general operations will probably be wound up by the end of this month. It is stated that the supplemental aid rendered in the Soudan Campaign by the British Red Cross Society has met with a very favourable impression in Egypt and the Soudan.

